# Aberrant brain entropy in posttraumatic stress disorder comorbid with major depressive disorder during the coronavirus disease 2019 pandemic

**DOI:** 10.3389/fpsyt.2023.1143780

**Published:** 2023-06-02

**Authors:** Shishun Fu, Sipei Liang, Chulan Lin, Yunfan Wu, Shuangcong Xie, Meng Li, Qiang Lei, Jianneng Li, Kanghui Yu, Yi Yin, Kelei Hua, Wuming Li, Caojun Wu, Xiaofen Ma, Guihua Jiang

**Affiliations:** ^1^The Department of Medical Imaging Guangdong Second Provincial General Hospital, Guangzhou, China; ^2^The Second School of Clinical Medicine, Southern Medical University, Guangzhou, China; ^3^The Department of Nuclear Medicine, Guangdong Second Provincial General Hospital, Guangzhou, China

**Keywords:** posttraumatic stress disorder, major depressive disorder, brain entropy, fMRI, resting-state

## Abstract

**Aim:**

Previously, neuroimaging studies on comorbid Posttraumatic-Major depression disorder (PTSD-MDD) comorbidity found abnormalities in multiple brain regions among patients. Recent neuroimaging studies have revealed dynamic nature on human brain activity during resting state, and entropy as an indicator of dynamic regularity may provide a new perspective for studying abnormalities of brain function among PTSD-MDD patients. During the COVID-19 pandemic, there has been a significant increase in the number of patients with PTSD-MDD. We have decided to conduct research on resting-state brain functional activity of patients who developed PTSD-MDD during this period using entropy.

**Methods:**

Thirty three patients with PTSD-MDD and 36 matched TCs were recruited. PTSD and depression symptoms were assessed using multiple clinical scales. All subjects underwent functional magnetic resonance imaging (fMRI) scans. And the brain entropy (BEN) maps were calculated using the BEN mapping toolbox. A two-sample *t*-test was used to compare the differences in the brain entropy between the PTSD-MDD comorbidity group and TC group. Furthermore, correlation analysis was conducted between the BEN changes in patients with PTSD-MDD and clinical scales.

**Results:**

Compared to the TCs, PTSD-MDD patients had a reduced BEN in the right middle frontal orbital gyrus (R_MFOG), left putamen, and right inferior frontal gyrus, opercular part (R_IFOG). Furthermore, a higher BEN in the R_MFOG was related to higher CAPS and HAMD-24 scores in the patients with PTSD-MDD.

**Conclusion:**

The results showed that the R_MFOG is a potential marker for showing the symptom severity of PTSD-MDD comorbidity. Consequently, PTSD-MDD may have reduced BEN in frontal and basal ganglia regions which are related to emotional dysregulation and cognitive deficits.

## 1. Introduction

Posttraumatic stress disorder (PTSD) and major depressive disorder (MDD) are common psychiatric mental disorders associated with the outbreak of the coronavirus disease 2019 (COVID-19); according to an epidemiological study, the prevalence of PTSD and depression was 21.94 and 15.97%, respectively (1). As a stressful event, the coronavirus disease 2019 (COVID-19) pandemic outbreak has had a critical impact on mental health and has been associated with both PTSD and depression (2–5). Therefore, unprecedented public health measures have been implemented to prevent the spread of the virus, as over half of the population was affected by this epidemic (6–8). Although home quarantine is an effective strategy to curtail virus transmission (9–11), it can have negative psychiatric effects simultaneously. Studies have reported the occurrence of negative psychological effects including PTSD, depression, anxiety, and insomnia during the home quarantine (12) and also showed that longer quarantine durations were associated with PTSD and depression (13–15).

PTSD is a highly comorbid disease, and epidemiological studies have reported that 52% of the patients with PTSD had comorbid depression (16–18). Recently, a genetic study indicated that PTSD is a subtype of MDD (19). In addition, studies have suggested that the co-occurrence of PTSD and MDD is linked with severe cognitive deficits, emotional symptoms, and a longer course than in patients with MDD or PTSD alone (20–22). For PTSD patients with comorbid depression, they require a larger dosage of psychotropic drugs compared to those with only PTSD (23). Although, a high incidence and severity of PTSD-MDD comorbidity have been established, the knowledge about its neurobiological mechanisms is limited.

The resting state functional magnetic resonance imaging (rs-fMRI) employs the blood oxygenation level-dependent (BOLD) signal to characterize the spontaneous activity of the brain (24). In addition, rs-fMRI is easy to implement and provides brain function time-resolved imaging at a relatively high spatial resolution compare to the EEG, making it a widely used tool for studying psychiatric diseases (25). Although rs-fMRI has been widely used in psychiatric disease, it is rarely applied in the study of PTSD-MDD comorbidity. A fMRI study suggesting that the subgenual anterior cingulate gyrus might be a potential neurobiological marker in distinguishing PTSD-MDD comorbidity from PTSD patients (26). Notably, current rs-fMRI studies have revealed the dynamic nature of the BOLD signals, which may reflect brain activity state or mental activity changes (27–30). Therefore, it may provide insight into the brain activity state in psychiatric disease by analyzing the dynamics regularity in fMRI time series. Wang et al. (31) developed a sample entropy (SampEn) toolbox to calculate the BEN maps based on the fMRI data. Although, the general algorithm for entropy needs a large dataset to precisely estimate the probability distribution function, whereas SampEn is an extension of Approximate Entropy and it showed preferable stability for different data lengths (32, 33). Therefore, SampEn is applicable for analyzing rs-fMRI data with an approximately small dataset.

Entropy indicates system irregularity. In the context of neural system time-based signals, it measures the irregularity of brain activity. Studies have suggested that the human brain should sustain entropy to maintain normal brain functioning (34, 35). Therefore, measuring the brain entropy (BEN) may be a physical means for characterizing brain activity state and its changes in psychological diseases. BEN has been characterized using electroencephalogram (EEG) data with relatively low spatial resolution (32, 36, 37). However, resting-state fMRI (rs-fMRI) provides an approximately high spatial resolution, that can be used for BEN mapping.

According to Wang et al. (31), over 1,000 healthy individuals’ SampEn maps showed significant lower entropy in the neocortex in compare with the rest of the brain. In addition, by comparing the changes in the BEN before and after caffeine intake in large samples of healthy subjects, Chang et al. (38) identified a significant caffeine-induced pattern of BEN increase in the whole brain. Furthermore, it has been shown in the study by Song et al. that BEN can provides features that cannot be fully described by other methods of resting-state brain activity, such as the cerebral blood flow (CBF) and the fractional amplitude of low-frequency fluctuation (ALFF) (39). Moreover, a current study also proposed the neurocognitive correlations of BEN in rs-fMRI (40). BEN alterations have been shown in various disorder conditions in clinical research, including schizophrenia (41, 42), MDD (43), attention deficit hyperactivity disorder (44), insomnia (45), and cocaine addiction (46). The BEN has been suggested as a potential biomarker for a number of psychiatric disorders. Studies on BEN represent a growing field on studying the dynamic nature of brain activities. we believe BEN can provide a novel insight into PTSD-MDD comorbidity.

The purpose of this study was to investigate the irregularity of the brain in patients with PTSD-MDD comorbidity by analyzing BEN in these patients and comparing them to traumatized controls. We observed that BEN had not been applied to the fMRI study PTSD or PTSD-MDD comorbidity; However, there are a few studies on MDD using the BEN. These studies propose that patients with MDD show BEN alterations in the brain regions associated with emotion regulation, such as the putamen and thalamus (47), and those vital for information processing, such as the medial orbital frontal cortex (43). Although these studies focused on MDD, previous studies have indicated that PTSD and MDD have quite a few overlapping symptoms, including anhedonia, sleep disturbance, and concentration difficulties (48). Therefore, we hypothesized that the BEN would differ between patients with comorbid PTSD-MDD and traumatized controls in the frontal regions and limbic systems, which are associated with information processing and emotion regulation.

## 2. Materials and methods

### 2.1. Participants

Participants include 33 drug-naive patients with PTSD-MDD comorbidity and 36 traumatized controls (TCs), who were enrolled at the Guangdong Second Provincial General Hospital in China and matched demographically. From December 2020 to October 2021, all the patients with PTSD-MDD were home-quarantined for 1 month or more during the COVID-19 epidemic, after which they received psychological consultation at the Guangdong Second Provincial General Hospital. The structured Mini-International Neuropsychiatric Interview for DSM-IV was administered by two experienced psychiatrists to assess PTSD-MDD comorbidity diagnosis. Subsequently, 33 patients were diagnosed with PTSD-MDD comorbidity. In addition, patients completed the PTSD Checklist Scale-Civilian (PCL-C) and the HAMD-24. TCs recruited from the local community were also quarantined for 1 month or more during the epidemic, and all of them showed no psychiatric symptoms after quarantine. The mental state of TCs subjects was also evaluated by two experienced psychiatrists. Lastly, each participant was matched according to sex, age, educational level, and hand dominance.

The inclusion criteria for patients with comorbid PTSD-MDD were as follows: (i) 1 month or more than 1 month of quarantine during the epidemic; (ii) met the criteria of DSM-IV (iii) PTSD Checklist-Civilian Version (PCL-C) score > 44 and HAMD score > 17; (iv) age > 18 years; and (iv) without a history of neurological disorders or psychiatric disorders. In contrast, the inclusion criteria for TCs were as follows: (i) age > 18 years; (ii) right-hand dominance; (iii) no psychiatric medications history; (iv) non-compliance with 3 Tesla fMRI safety standards. However, the groups did not differ in age or sex (Table 1). Furthermore, permission to conduct this study was granted by the ethics committee of Guangdong Second Provincial General Hospital. All the participants provided written informed consent in agreement with ethical approval from the Guangdong Second Provincial General Hospital committee.

**Table 1:** Demographic and clinical data.

Variables (mean ± SD)	PTSD-MDD	TCs	*p-*value
Sex (M/F)*	12/21	15/21	0.203
Age (years)	27.3 ± 9.47	25.4 ± 7.11	0.357
Education (years)	12.8 ± 3.05	12.8 ± 2.93	0.660
CAPS score	52.70 ± 7.38	6.58 ± 4.53	<0.0000
PCL-C score	49.30 ± 5.28	5.25 ± 3.41	<0.0000
HAMD-24 score	22.88 ± 4.57	3.36 ± 2.10	<0.0003

*For gender *p* value was calculated using the Chi-square (*χ*^2^) test; M/F, Male/Female; CAPS, Clinician-Administered PTSD Scale for DSM-IV; PCL-C, The PTSD Checklist-Civilian Version; HAMD-24, Hamilton Depression Scale-24 items; SD, Standard Deviation; PTSD-MDD, Posttraumatic Stress Disorder and Major Depression Disorder Comorbidity; TCs, Traumatized Controls.

### 2.2. Mental status assessment

The PTSD-MDD comorbidity diagnosis was determined according to the DSM-IV diagnostic criteria. Before undergoing the fMRI scanning, all the PTSD-MDD comorbidity patients were screened with the CAPS, PCL-C, and HAMD-24 to estimate the severity of the symptoms. Further structured clinical interview was conducted to assess other psychiatric comorbidities.

### 2.3. fMRI procedures

Scans were obtained in a single 3.0-T Philips MR scanner (Ingenia; Best, The Netherlands) equipped with a 32-channel head coil. The datasets include T2-FLAIR images, 3D T1-weighted images and gradient echo-planar images (EPI). The T2-FLAIR images were obtained to detect the participants with any brain lesions. The EPI data were acquired in an interleaved order to measure BOLD signal, the data were scanned approximately AP-PC line with the following parameters: repetition time/echo time (TR/TE) = 2,000/30 ms; matrix = 64 × 64, field of view (FOV) = 230 mm × 230 mm, flip angle (FA) = 90°, slice thickness = 3.6 mm slice, number of slices = 33, slice gap = 0.6 mm, 250 volumes were acquired within 500 s. 3D T1-weighted structural images were acquired for each participant with TR/TE = 25/4.1 ms; FA = 30°, matrix = 256 × 256, FOV = 230 mm × 230 mm, slice thickness = 1.0 mm; number of slice = 160, slice gap = 0. During the scan, the participants wore headphones to reduce noise, and placing soft pads on the sides of the head to minimize the head movement. All the participants were instructed to lie still, stay awake, and think of nothing in particular.

### 2.4. Data processing and BEN calculations

#### 2.4.1. Preprocessing

MRI data were preprocessed using the Statistical Parametric Mapping version 12 (SPM12) software^1^. (1) Before preprocessing, the first 10 EPI volumes of each subject’s data were discarded to allow for image intensity to reach stable state. (2) The remaining volumes were performed with correction of intra-volume time delay using the middle slice as reference and inter-volume head motion using the first volume as the reference. (3) 3D T1-weighted images were registered into Montreal Neurological Institute (MNI) space, gray matter, white matter and cerebrospinal fluid maps were segmented and generated during this process. (4) The EPI images were spatially co-registered with the 3D T1-weighted structural images as mentioned above and resampled into 3 mm isotropic voxels. (5) We used the mean framewise displacement (FD) Jenkinson as the head motion reference standard. We eliminated the participants with motion (mean FD Jenkinson) > 2 × Standard Deviation (SD) above the group mean motion as we did in our previous study (49). (6) Temporal nuisance signals were regressed out including the head motion parameters (Friston 24 model), the cerebrospinal fluid signal, and white matter signal, global signal was not regressed out (50). Subsequently, linear detrend and bandpass filtering (0.01–0.08 Hz) were performed to minimize the low-frequency drift and high-frequency physiological noise. Finally, the data were spatially smoothed with a 10 mm full-width at half-maximum Gaussian kernel.

#### 2.4.2. BEN calculation

A combination of home-designed Matrix Laboratory (MATLAB) code and the Brain Entropy Mapping toolbox (BENtbx) developed by Wang et al. (31) were used to calculate the sample entropy for each voxel after image preprocessing (33). SampEn is an extension of Approximate Entropy (ApEn), its determined from the temporal coherence of a time-series. SampEn is calculated the “logarithmic likelihood” that a small window (length “m”) of the data “matches” with other windows whether it will still “match” the other windows if the window length increases by 1 (length “m + 1”). The “match” depends on the tolerance threshold value <“*r*” times SD of the entire time series. Details of BEN calculation was described in the original BENtbx paper (31). Same as previous studies the “*m*” equal to 3 and the “*r*” equal to 0.6 and the threshold is *r* * SD (39).

#### 2.4.3. Statistical analysis

A two-sample t-test was used to compare the differences in age and education level, While, a chi-square test was used to compare the gender composition between the two groups. A two-sample t-test was used to compare the differences in the brain entropy between the PTSD-MDD comorbidity group and TC group by using the DPABI toolbox,^2^ covariates included age, sex, and educational level. The false discovery rate (FDR) correction was used for the multiple comparison correction, and the significance level was set at *p* < 0.05. Therefore, to explore the relationship between the average BEN values of the ROIs and clinical indicators, we performed general linear models with the CAPS, PCL-C, HAMD-24, and BEN values from the clusters that showed significant group differences as independent variables, and age, gender, educational level, and head motion (mean FD Jenkinson) as covariates. Statistical analysis was performed using the Statistical Package Social Sciences (SPSS) software version 2.3, with a significance threshold set at *p* < 0.05.

## 3. Results

### 3.1. Demographic and clinical tests

There were no significant inter-group differences in terms of age, sex, or education level (all *p* > 0.05). However, in neuropsychology assessments, there were significant differences between patients with PTSD-MDD and the TCs in CAPS, PCL-C, and HAMD-24. Table 1 shows the demographic and neuropsychological assessment results.

### 3.2. Altered BEN values in patients with PTSD-MDD comorbidity

**Figure 1:**
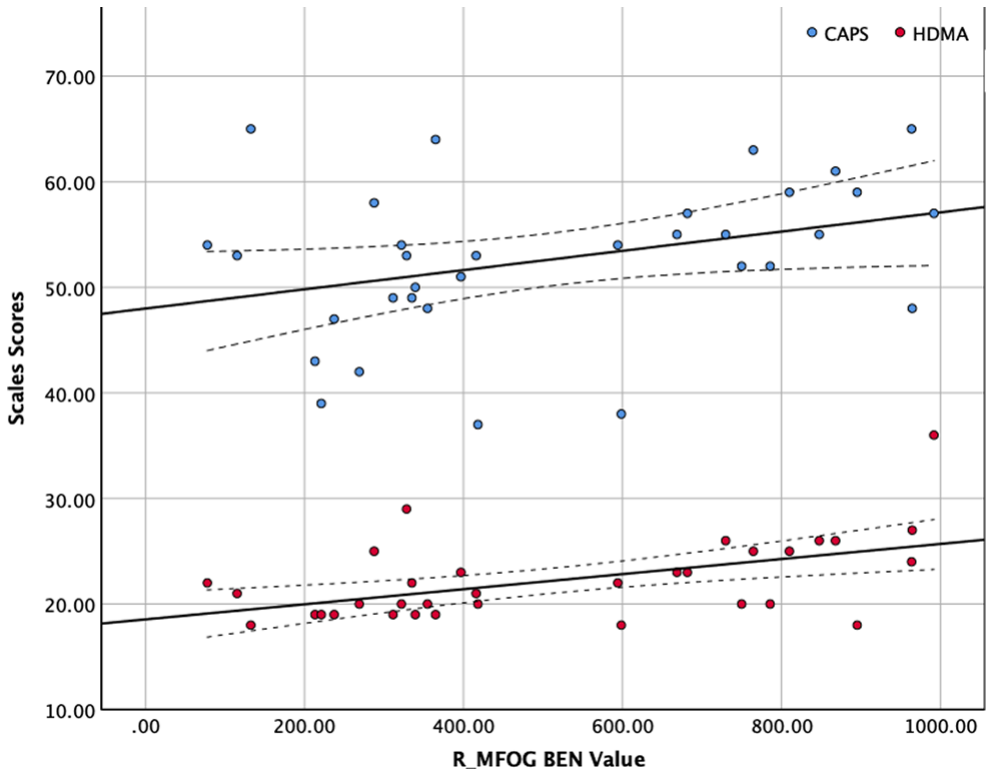
Two-sample *t*-test performed to test the differences in the brain entropy (BEN) maps between the posttraumatic stress disorder and major depressive disorder (PTSD-MDD) group and the traumatized controls (TCs) at each voxel. In addition, the false discovery rate (FDR) tests were performed for multiple comparison corrections (*p* < 0.05), PTSD-MDD group showed significantly reduced BEN value in the right middle frontal orbital gyrus (R_MFOG), left putamen, and the right inferior frontal gyrus, opercular part (R IFOG).

Figure 1 shows the results of the group-level analysis. The results showed that patients with PTSD-MDD had significantly decreased BEN values in the right middle frontal gyrus orbital part (R_MFOG), left putamen and right inferior frontal gyrus, opercular part (R_IFOG), after FDR correction compared to TCs (Table 2; Figure 1).

**Table 2:** Comparison of SampEn between PTSD-MDD and HC.

Brain region	Cluster size	MNI coordinate			AAL	BA	Peak *t* value
		*X*	*Y*	*Z*			
R MFOG	570	34	38	−16	Frontal_Mid_Orb_R	BA11	−4.5913
L Putamen	178	−16	6	−6	Putamen_L		−4.6276
R IFOG	313	62	16	2	Frontal_Inf_Oper_R	BA47	−4.6032

L, left; R, right; MNI, Montreal Neurological Institute; AAL, Anatomical Automatic Labeling; BA, Brodmann Area; MFOG, Middle Frontal Orbital Gyrus; IFOG, Inferior Frontal Gyrus, Opercular part.

### 3.3. Correlations between BEN values of abnormal regions and CAPS score, HAMD score

**Figure 2:**
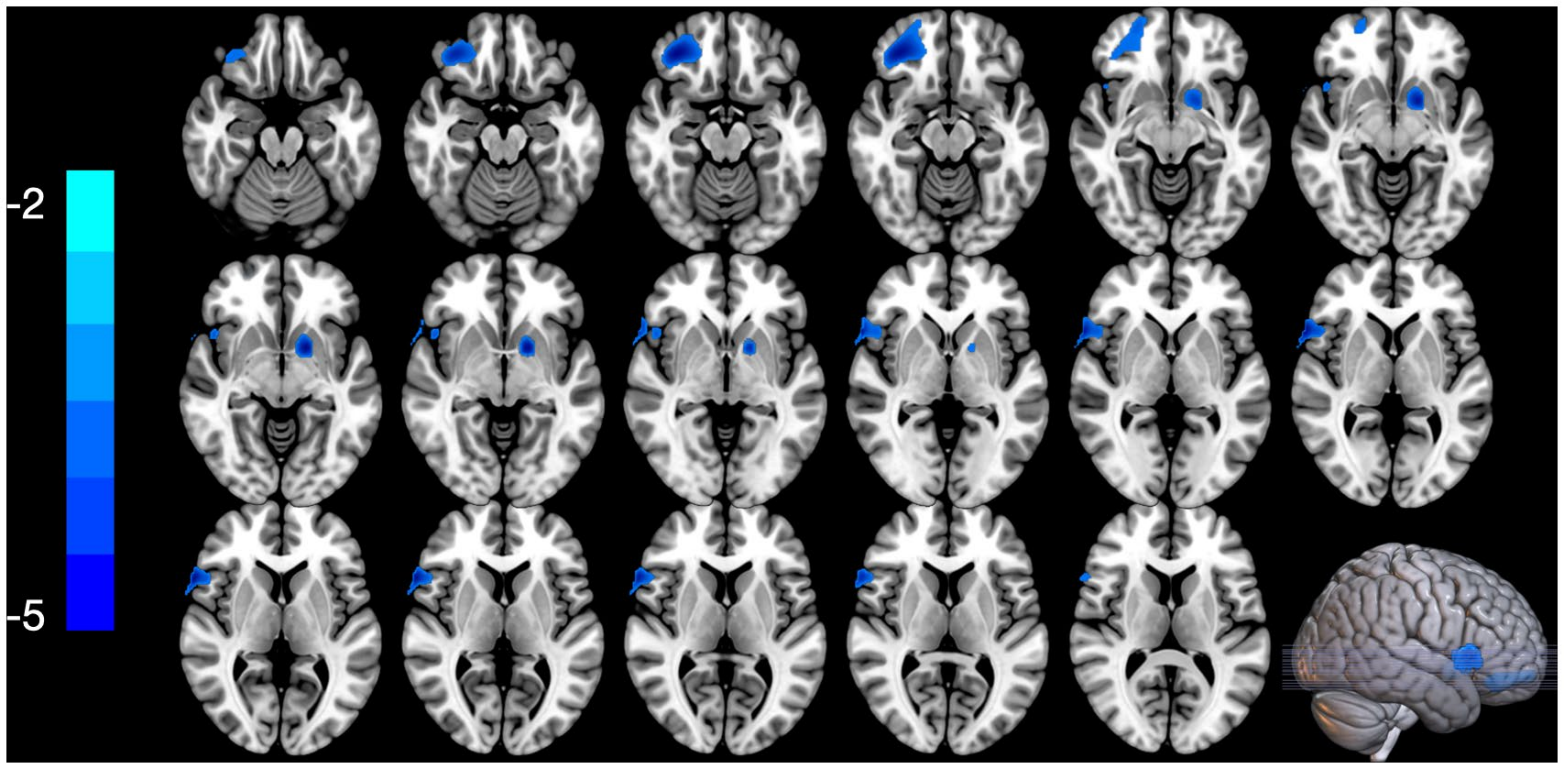
Correlation between right middle frontal orbital gyrus (R_MFOG) brain entropy (BEN) values and Clinician-Administered PTSD Scale (CAPS) and Hamilton Depression Scale-24 items (HAMD-24) scores. The BEN values of the R_MFOG were positively correlated with CAPS scores and HAMD-24 scores (CAPS: *r* = 0.366, *p =* 0.036; HAMD-24: *r* = 0.479, *p =* 0.002).

The BEN values and clinical scales were correlated using Spearman’s correlation because of the limited sample size of this study (see in Figure 2). Figure 2 shows that R_MFOG BEN in was positively correlated with HAMD-24 (*r* = 0.479, *p =* 0.005) and CAPS (*r* = 0.366, *p* = 0.036).

## 4. Discussion

This study examined the BEN maps in PTSD-MDD by comparing the BEN maps in the PTSD-MDD and TCs. The following are two major findings derived from these results: (1) Patients with PTSD-MDD showed lower BEN in the R_MFOG, left putamen, and R_IFOG; and (2) BEN values in the R_MFOG was positively correlated with HAMD-24 and CAPS. These findings support our hypothesis that patients with PTSD-MDD show significant differences in the BEN in the frontal regions and limbic system.

The reduced BEN found in the R_MFOG during the resting state in patients with PTSD-MDD is partially consistent with those of previous studies (51–53). However, a resting-state perfusion study showed a reduced cerebral blood flow (CBF) in the R_MFOG, and the reduced CBF in the R_MFOG was negatively correlated with PTSD severity (51). Our study also showed reduced BEN in the R_MFOG, but the reduced BEN was positively correlated with PTSD severity and depression severity. A previous study showed BEN-CBF correlation especially in the MFOG and inferior temporal cortex (39), since the BOLD signal is mostly contributed by the CBF (54). These studies may explain the concordance between our results and the CBF results. Interestingly, a previous study indicated that BEN is mostly independent of CBF; they found that caffeine induced whole brain CBF decrease, but a large portion of BEN increased including the prefrontal cortex (38). These results suggest that caffeine induced CBF decrease, but caffeine induced BEN increase may suggest enhanced cognition in the subject. Electroencephalographic (EEG) studies have also indicated that BEN is associated with cognition and emotion (55, 56). Accordingly, we assumed that CBF reduction and BEN reduction in PTSD are independent, and that the correlation between PTSD severity and BEN may be related to emotional and cognitive symptoms.

Functional imaging studies of PTSD have reported hypoactivity in the ventromedial frontal cortex including in the R_MFOG (57, 58). Memory encoding and retrieval are affected by reduced activity of prefrontal cortex, resulting in difficulty in restructuring their traumatic memory in PTSD patients (59). The R_MFOG usually shows reduced connection in the EEG and fMRI studies, suggesting that the reduced connectivity in the R_MFOG might be related to dysregulation of the default mode network or self-referential processing process in patients with PTSD (60, 61). Furthermore, several neuroimaging findings propose that combined with the limbic system the orbital and medial frontal cortex is critical in the MDD aberrant network (62–64). Previous studies have suggested that the dysfunctional orbital frontal cortex is associated with suicidal symptoms, as it plays a key role in decision-making (65). On the other hand, the orbital frontal cortex is also involved in stressful events and has increased functional connectivity (FC) especially in self-referential processes, that can cause depression and frustration (66, 67). Accordingly, we believe the decreased BEN in the R_MFOG is associated with depression symptoms, and the BEN may reflect the severity of MDD.

Conversely, the reduced R_IFOG in this study are partly supported by a previous BEN study on MDD. A previous study found a decrease in the BEN value of the R_IFOG. However, they also observed a negative correlation between the HAMD-24 and the BEN value of the inferior frontal region (68). In addition, a previous task-based fMRI study reported increased FC in IFOG (69), proposing that the increased connectivity is associated with the fronto-temporo-parietal network, and the altered connectivity in this network may result from a compensatory mechanism, which is important for emotion regulation (70). There is evidence that IFOG is involved in cognition as well as emotional processing, including the association between MDD patients and the preference for processing negative information (71). A voxel-based morphometry study indicated that patients with MDD and patients with PTSD-MDD showed smaller volumes in the IFOG than in the healthy controls, and that the IFOG volume was normalized after medication and the depressive symptoms were also mitigated (72). Therefore, we assume that the reduced BEN in the R_IFOG might be associated with depression symptoms, and that abnormalities in the IFOG might indicate the depressed patients with anxiety comorbidities. Several structural studies have demonstrated that the reduced inferior frontal cortex in depressed patients is comorbid with anxiety symptoms (73, 74). In line with these studies, our findings provide evidence of the functional irregularity in the R_IFOG in patients with PTSD-MDD comorbidity.

The finding of reduced BEN in putamen is partly in agreement with a previous study of BEN in patients with MDD (68). Not only that, but a previous fMRI study showed a decreased FC between the basolateral amygdala and putamen in PTSD-MDD comorbidity versus PTSD alone. Furthermore, they found a negative correlation between the basolateral amygdala-putamen FC and the HAMD-24 scores (75). These results indicate that the FC difference between the PTSD-MDD and PTSD groups may be more closely associated with MDD symptoms. Although we could not find correlations between the HAMD-24 scores and the BEN value in the putamen, several studies have suggested that putamen is associated with depressive symptoms (76, 77). In addition, a previous MDD study also showed weaker FC between the amygdala and the cortico-striatal-pallidal-thalamic circuit, which maintains information in working memory (78). There is a correlation between working memory, as a main cognitive deficit, and PTSD (79, 80). Accordingly, we believe that the reduced BEN in the putamen may meditate an impaired working memory in patients with comorbid PTSD-MDD.

This study had some limitations. First, this study lacked a PTSD-only group or MDD-only group, making it impossible to evaluate whether patients with PTSD-only patients or MDD-only patients had different BEN than those with PTSD-MDD. Second, as all our patients were exposed to trauma, most people in the urban areas had home-quarantine experience, and the social panic following the outbreak is an important traumatic factor. Third, the small sample size may have resulted in insufficient statistical power. Fourth, although previous studies have indicated the BEN reflects the human brain complexity of human brain, the exact neurophysiological basis remains unclear, limiting the interpretation of our findings.

In conclusion, this study revealed a decreased BEN in patients with PTSD-MDD who were home-quarantined for 1 month or more during the COVID-19 epidemic. Therefore, these results not only provide evidence for the BEN research on PTSD-MDD comorbidity but also provide evidence that pandemic-related psychiatric disease affects the brain irregularities in brain activity. Furthermore, the brain regions that show significant BEN alterations agree with previous fMRI studies, and correlations were established between the BEN in the right MFOG and the symptom severity (CAPS, HAMD-24), suggesting that MFOG is a potential marker for revealing the mechanism of PTSD and MDD and may inform future clinical interventions.

## Data availability statement

The original contributions presented in the study are included in the article/supplementary material, further inquiries can be directed to the corresponding authors.

## Ethics statement

The studies involving human participants were reviewed and approved by the Ethics Committee of Guangdong Second Provincial General Hospital. The patients/participants provided their written informed consent to participate in this study.

## Author contributions

All authors listed have made a substantial, direct, and intellectual contribution to the work and approved it for publication.

## Funding

This study was funded by grants from the National Natural Science Foundation of China (Grant Numbers: U1903120, 81771807, 81901729, and 82001792) and the Science and Technology Planning Project of Guangzhou (Grant Number: 202002030234) and the Science Foundation of Guangdong Second Provincial General Hospital (No. 3D-A2021009).

## Conflict of interest

The authors declare that the research was conducted in the absence of any commercial or financial relationships that could be construed as a potential conflict of interest.

## Publisher’s note

All claims expressed in this article are solely those of the authors and do not necessarily represent those of their affiliated organizations, or those of the publisher, the editors and the reviewers. Any product that may be evaluated in this article, or claim that may be made by its manufacturer, is not guaranteed or endorsed by the publisher.

## Footnotes

^1^ http://www.fil.ion.ucl.ac.uk/spm

^2^ http://rfmri.org/dpabi
